# Intracellular Microbiome Profiling of the *Acanthamoeba* Clinical Isolates from Lens Associated Keratitis

**DOI:** 10.3390/pathogens10030266

**Published:** 2021-02-25

**Authors:** Yu-Jen Wang, Sung-Chou Li, Wei-Chen Lin, Fu-Chin Huang

**Affiliations:** 1 Institute of Basic Medical Sciences, College of Medicine, National Cheng Kung University, Tainan 70101, Taiwan; naohito123@gmail.com; 2 Genomics and Proteomics Core Laboratory, Department of Medical Research, Kaohsiung Chang Gung Memorial Hospital and Chang Gung University College of Medicine, Kaohsiung 83325, Taiwan; raymond.pinus@gmail.com; 3 Department of Parasitology, College of Medicine, National Cheng Kung University, Tainan 70101, Taiwan; 4 Department of Microbiology and Immunology, College of Medicine, National Cheng Kung University, Tainan 70101, Taiwan; 5 Department of Ophthalmology, National Cheng Kung University Hospital, College of Medicine, National Cheng Kung University, Tainan 70403, Taiwan

**Keywords:** *Acanthamoeba*, keratitis, metagenomics, contact lens

## Abstract

*Acanthamoeba* act as hosts for various microorganisms and pathogens, causing *Acanthamoeba* Keratitis (AK). To investigate the association between endosymbionts and AK progression, we performed a metagenomics study to characterize the intracellular microbiome from five lenses associated with AK isolates and standard strains to characterize the role of ocular flora in AK progression. The used clinical isolates were axenic cultured from lenses associated with AK patients. AK isolates and standard controls such as 16S ribosomal RNA sequencing techniques were used for analysis. The microbiome compositions and relative abundance values were compared. The orders of *Clostridiales* and *Bacteroidales* presented major populations of intracellular microbes belonging to all isolates. Comparison of the different source isolates showed that most of the abundance in keratitis isolates came from *Ruminococcus gnavus* (121.0 folds), *Eubacterium dolichum* (54.15 folds), *Roseburia faecis* (24.51 folds), and *Blautia producta* (3.15 folds). Further analysis of the relative abundance data from keratitis isolates showed that *Blautia producta* was positively correlated with the disease course. In contrast, *Bacteroides ovatus* was found to be abundant in early-stage keratitis isolates. This study reveals the abundant anaerobic Gram-positive rods present in severe keratitis isolate and characterize the association between *Acanthamoeba* and ocular flora in AK progression.

## 1. Introduction

*Acanthamoeba* is a free-living amoebae (FLA) that can be found in moist environments, such as in tap water and human nasopharyngeal mucosa [1,2]. The major pathogenic strains include *Acanthamoeba castellanii* and *Acanthamoeba polyphaga*. Diseases in Acanthamebiasis contain *Acanthamoeba* keratitis (AK) and Granulomatous amoebic encephalitis (GAE). The protozoa obtain food sources from surrounding microorganisms via phagocytosis. Nevertheless, several bacteria can escape and latently inhabit amebae, including ocular surface flora.

The ocular surface harbors a variety of microbes due to continuous exposure to the environment [3]. The ocular commensals play a crucial role in defending against pathogens and maintaining ocular immunity [4]. A recent study demonstrated that the *Acanthamoeba* characteristics of phagocytosis alter the surrounding microorganism distribution, thereby causing complications [5,6]. *Acanthamoeba* can host and protect microorganisms, including pathogenic bacteria, fungi, and viruses [7]. However, some microorganisms can survive digestion and multiply within hosts, which are called endosymbionts. The association between endosymbionts and their hosts can be either temporary or steady. Since such “Trojan horses” can escape the intracellular killing of *Acanthamoeba*, these microbes could be protected from harmful environments, such as ocular immune killing [8]. Previous studies demonstrated that *Pseudomonas aeruginosa* is an important endosymbiont of *Acanthamoeba* and that it usually infects concurrently with AK [9]. Some pathogens such as *Mimivirus* can acquire genes from the ameba host [10]. These genes can be exchanged and subsequently produced by endosymbionts [11], suggesting the role of *Acanthamoeba* in microorganism evolution. In addition, several studies have indicated that endosymbionts can enhance the pathogenicity of *Acanthamoeba* [12,13]. In vitro and in vivo studies of cytopathic effect (CPE) showed significant increases in endosymbiont-infected *Acanthamoeba* [14,15]. Therefore, the intracellular microbiome profiling of *Acanthamoeba* clinical isolates might be a potential entry point for a pathogenicity differentiation between *Acanthamoeba* isolates.

Most AK patients who have been wearing contact lenses (CLs) over a long period have a high risk of contracting AK. According to previous studies, the long-term wearing of CLs, not only affects the eye’s health but also interferes with the ocular surface microbiota distribution through oxygen deprivation [16,17]. Recent studies have demonstrated the relationship between the dysbiosis of the ocular surface microbiome and several conditions, such as dry eyes [18] and the wearing of CLs [19]. Therefore, the use of CLs might create a sealed hunting ground for *Acanthamoeba*, which could lead to depletion of probiotics. Nevertheless, some studies have analyzed the microbiota in CLs users [20,21], whereas the characterization of the microbiome with *Acanthamoeba* involvement is less reported. As most AK isolates are related to CLs use [22,23], investigation of the association between the microbiome and clinical AK isolates would be necessary for disease diagnosis and prognosis.

In this study, bacterial 16S ribosomal RNA sequencing techniques were performed to profile the intracellular microbiome of phagocytic microbes in various CL-associated AK isolates in order to characterize the association between *Acanthamoeba* and the intracellular microbiome in AK progression in a hypoxic environment.

## 2. Results

### 2.1. The Isolates Were Collected from Various Conditions

To determine the intracellular microbiome in the *Acanthamoeba* spp., we collected five clinical and two standard isolates. All isolates were cultured until normalization. The clinical isolates belonged to *Acanthamoeba castellanii* strain T4 and contained axenic culture from *Acanthamoeba* keratitis (AK) patients, who were assessed at Cheng Kung University Hospital, Taiwan. All clinical isolates were collected from patients who had lenses as a risk factor [24]. Standard isolate ATCC-30010 was collected from environmental soil in the United State and isolate ATCC-50492 was isolated from a keratitis patient from India, as described by the official website of ATCC. The NCKUH-A, B, and C strains were isolated from patients receiving medical treatment for early keratitis. The NCKUH-D and H strain patients had received surgery for late keratitis (Table 1). The isolate profiles were used as analytic factors in this study.

### 2.2. Specific Bacterial 16S Primers Had Detected the Intracellular Microbes in All Isolates

**Figure 1:**
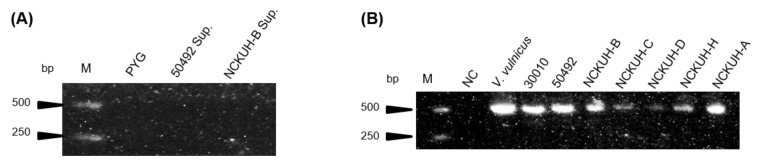
Amplification of PCR cultured medium extracts and *Acanthamoeba* DNA samples using bacterial 16S primers. (**A**) Isolate ATCC-50492 and NCKUH-B cultured medium extracts were amplified by bacterial 16S primers and sterile peptone-yeast extract-glucose (PYG) medium as a negative control. (**B**) *Acanthamoeba* DNA samples amplified with bacterial 16S primers and PCR products presented at 550 bps. *Vibrio vulnicus* was used as positive control and distillation–distillation H_2_O was used as a negative control for the 16S primers. M: marker; NC: negative control

*Acanthamoeba* spp. DNA was extracted from the axenic cultures and the bacterial specific 16S primer pairs were used to pre-examine the endosymbionts using RT-PCR. No contamination was detected in this study after the screening of isolate ATCC-50492 in cultured medium and NCKUH-B using PCR detection (Figure 1A). PCR electrophoresis showed only a single pattern belonging to the isolates (Figure 1B), which indicated that the designed primer pairs showed specificity and that the isolates harbored phagocytic intracellular microbes.

### 2.3. Clostridiales and Bacteroidales Are the Major Intracellular Microbes of Isolates

**Figure 2:**
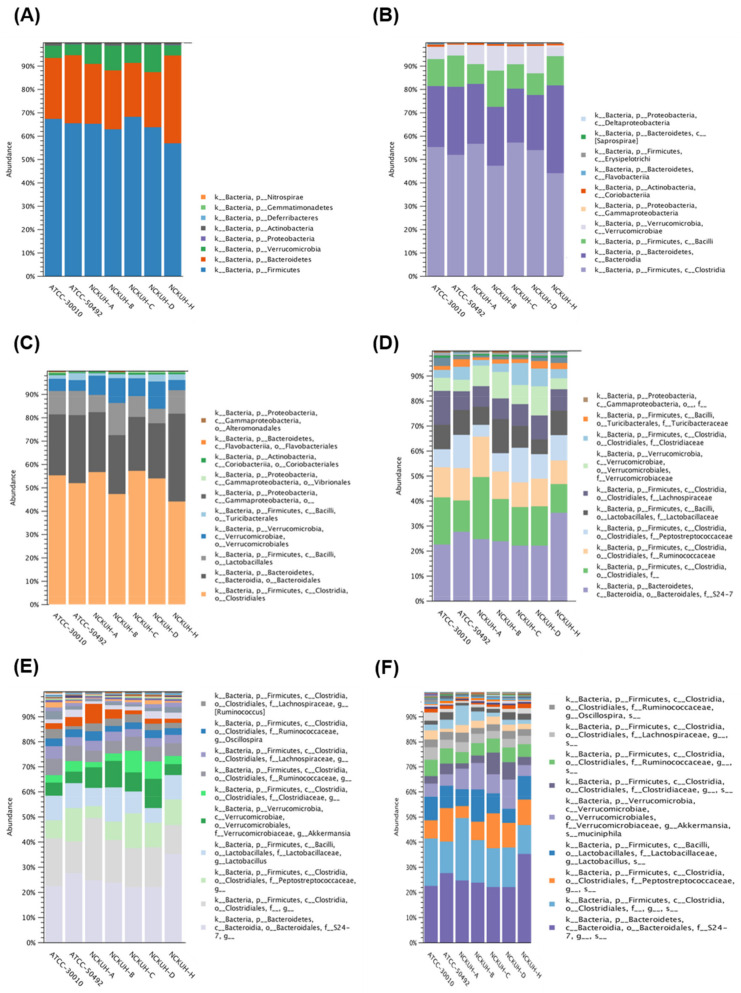
16S rRNA gene sequencing of *Acanthamoeba castellanii* isolates. The intracellular microbes content in the individual strains of *A. castellanii*. The comparison data from phylum to species are shown in (**A**–**F**), respectively.

Regarding the 16S sequencing data from individual isolates, there were no unique differences present in all isolates. All isolates had major populations of the orders *Clostridiales* and *Bacteroidales* (Figure 2).

**Figure 3:**
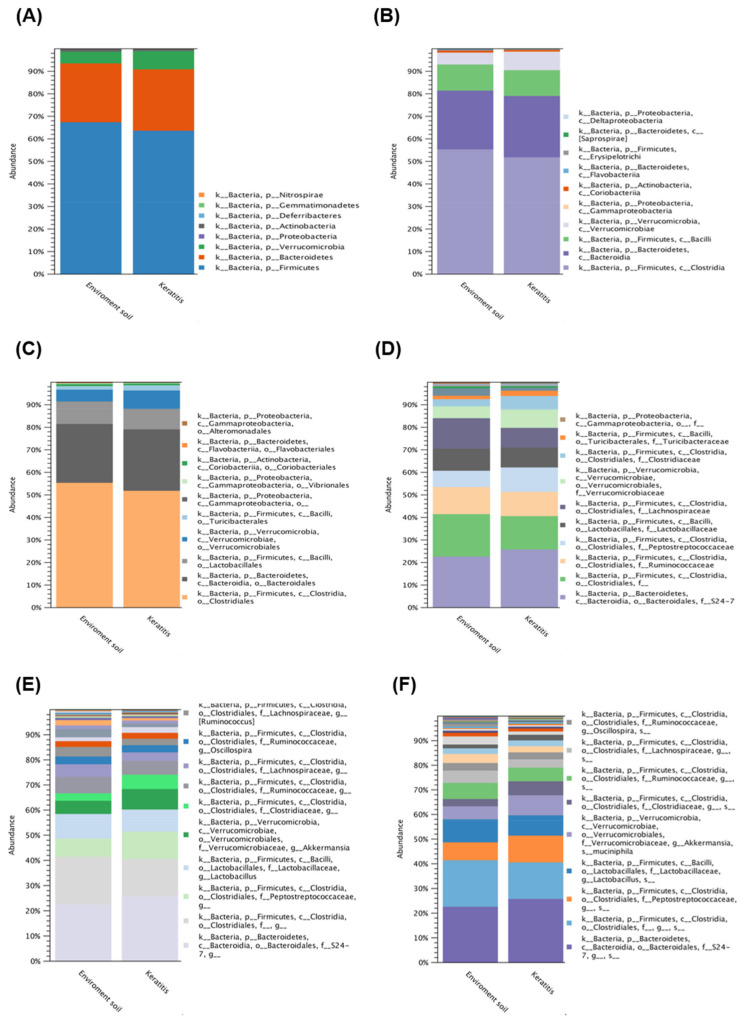
16S rRNA sequencing data comparison of the isolated resource. The intracellular microbes content in the isolates source of soil and *Acanthamoeba* keratitis (AK) patients. The comparison data from phylum to species are shown in (**A**–**F**) respectively.

**Figure 4:**
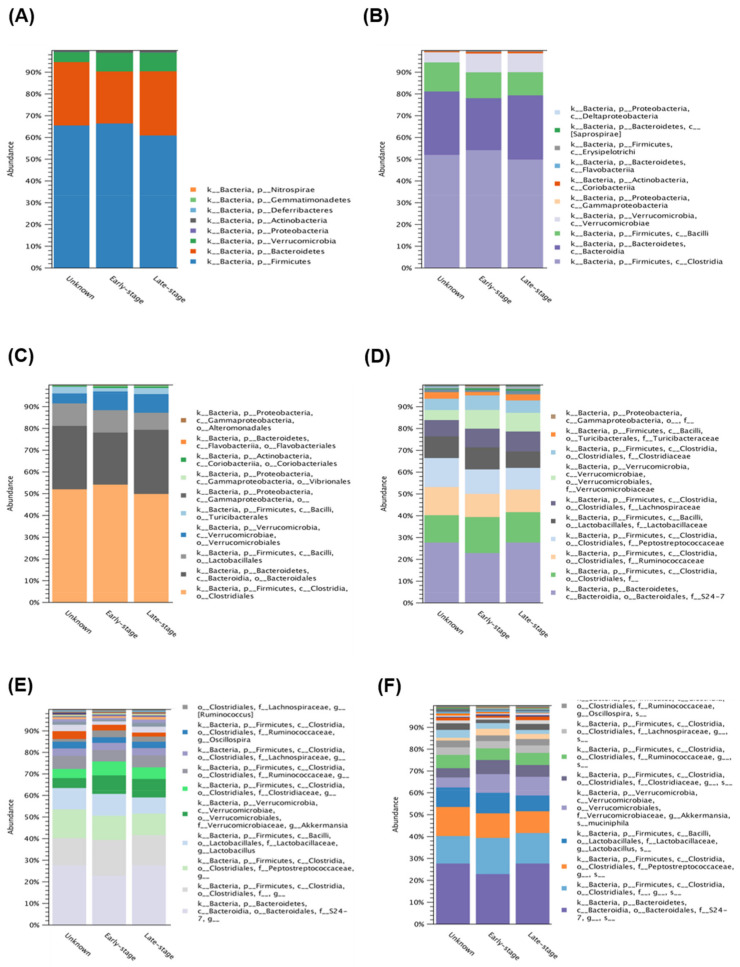
16S rRNA sequencing data comparison of different progression in *Acanthamoeba* keratitis. The intracellular microbes content in the progression of AK isolates. The comparison data from phylum to species are shown in (**A**–**F**) respectively.

To further investigate the diversity between various *Acanthamoeba* spp. isolates, we compared different groups, which are described in Table 1. Comparing various isolates, either isolate ATCC-30010 from the soil source or the others from keratitis patients, all contained *Clostridiales* and *Bacteroidales* as the major populations of intracellular microbes (Figure 3). The same populations also appeared in the disease progression comparison (Figure 4). However, the two major groups could not be defined at the genus or species level owing to technical obstacles in the 16S ribosomal RNA sequencing comparison.

### 2.4. Blautia Product Showed Abundance in the Isolates of Severe *Acanthamoeba* Keratitis

**Figure 5:**
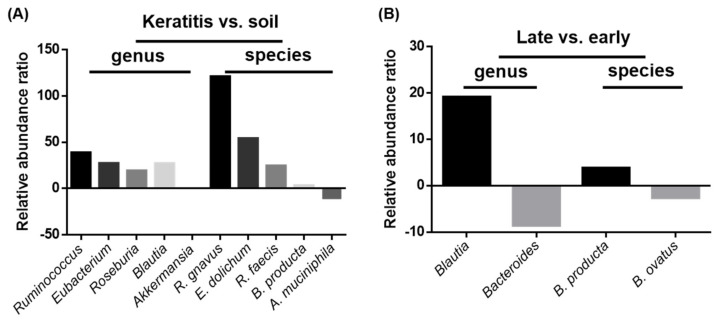
Intracellular microbe relative abundance ratios of keratitis vs. soil source and late vs. early keratitis comparisons at genus and species levels. (**A**) Microbe relative abundance ratios of keratitis vs. soil source at genus and species levels. (**B**) Microbe relative abundance ratios of late vs. early keratitis patients at genus and species levels. The compared microbes are displayed on the X axis and were validated by identification in both genus and species.

To investigate the microbiome diversity with *Acanthamoeba* spp. pathogenicity, we compared the relative abundances from the different groups of gene expression databases. Interestingly, the clinical keratitis isolates showed a greater abundance of *Ruminococcus gnavus* (121.0 folds), *Eubacterium dolichum* (54.15 folds), *Roseburia faecis* (24.51 folds), and *Blautia producta* (3.15 folds) than those from soil sources. Although *Akkermansia muciniphila* was abundant in soil-source ameba (11.08 folds), the relative abundance was not different in the genus validation. These abundant intracellular microbes were also presented in genera of *Ruminococcus* (38.79 folds), *Eubacterium* (27.27 folds), *Roseburia* (19.16 folds), and *Blautia* (27.01 folds) during the validated comparison (Figure 5A). Furthermore, the comparison of intracellular microbe abundance in diverse keratitis progression stages showed that late-stage keratitis isolates had more *Blautia producta* (3.81 folds). This significant difference was also observed during the genus validation of *Blautia* (19.15 folds). The early-stage keratitis isolates harbored abundant microbes of *Bacteroides ovatus* (2.60 folds), as proven by genus validation (8.55 folds) (Figure 5B).

## 3. Discussion

Free-living *Acanthamoeba* hosts harbor various intracellular microbes, which have been known for a long time [25]. In the present study, we analyzed five clinical isolates and two standard isolates to reveal the abundant microbes: *Ruminococcus* and *Blautia*. Of a total of seven *Acanthamoeba* isolates, six were obtained from lens-associated AK patients and one from a soil source which was considered as a non-disease-related strain. Of the five clinical isolates, three (NCKUH-A, B, and C) were isolated from patients who received medically successful AK treatment, whereas two (NCKUH-D and H) were from patients who finally had surgery owing to poor progression of AK. The intracellular microbiome content conversion profiles in different groups showed no obvious differences. However, the intracellular microbe relative abundance profiles from gene expression databases showed interesting diversity. The clinical isolates from lens-associated keratitis had a greater abundance of intracellular microbes of *R. gnavus*, *E. dolichum*, *R. faecis*, and *B. producta* than non-disease related strains. In contrast, the NCKUH-D and H isolate from medical failure cases had more *B. producta* and less *B. ovatus* in comparison with medical success isolates. Hence, the intracellular microbe relative abundance data suggested that the anaerobic Gram-positive bacillus *Blautia producta* might play roles in *Acanthamoeba* spp. pathogenicity in lens-wearing patients.

*B. producta* was described in 1941 [26] and is commonly found in human feces. It has the capacity for rapid growth at the expense of carbon monoxide (CO) through anaerobic digestion [27]. *B. ovatus* was first reported in 1933 [28] and provides an inherent biosafety feature in the human gut [29]. However, *B. producta* is Gram-positive and *B. ovatus* is Gram-negative. According to previous studies, human ocular microbiota was characterized to be commonly identified in Gram-positive bacteria [30], while *Acanthamoeba* apparently prefer Gram-negative bacteria [31]. Previous studies had demonstrated that amoebae prefer certain species of bacteria as a food source, such as *P. aeruginosa* and *S. marcescens* [32], but not *S. epidermidis* [33,34]. It was presumed that the gram-positive cell wall was thick and hard to be digestion for *Acanthamoeba*. Therefore, the early-stage keratitis isolates harbored abundant Gram-negative bacteria, with *B. ovatus* suspected as the early food source during initial infection. Notably, the relative abundance of *B. ovatus* was significantly higher in patients with atopic dermatitis as an immune disorder [35], whereas *B. producta* had demonstrated prevention of Vancomycin-resistant Enterococcus (VRE) colonization [36]. These results suggest the importance of *Acanthamoeba* ingestion of bacteria, which might lead to secondary inflammation or infection due to ocular surface dysbiosis.

Additionally, both *B. producta* and *B. ovatus* are anaerobic microbes, which were not the major populations of ocular microbiota. This was probably due to vigorous reproduction on the ocular surface, which made a hypoxic environment, leading to lens obstruction. Although several diseases can lead to ocular hypoxia, including diabetes, herpes simplex virus infection, dry eye syndrome, and limbal stem cell deficiency [37], lens use has become the common factor in induced ocular hypoxia, due to the increasing use of CLs [38]. Previous studies had demonstrated that amoebae containing endosymbionts had more high pathogenicity than endosymbiont-free amoebae. Endosymbionts may influence *Acanthamoeba* pathogenicity, virulence, or susceptibility to drugs [39]. However, the ingested microorganisms might play roles in the protection of the cornea. Recent studies on ocular microbiota found that common genera, such as *Staphylococci* and *Streptococcus*, can stimulate ocular immunity as a natural barrier on the corneal surface [40]. Under hypoxia and less remaining microbiota condition, AK could be predicted to poor progression and *Acanthamoeba* isolates were consider as high pathogenicity. Therefore, the analysis of endosymbionts in *Acanthamoeba* might be beneficial for AK patient prognosis.

In conclusion, these findings indicate that the types of intracellular microbes were associated with AK progression, while the abundant anaerobic bacteria in the keratitis isolates were associated with CLs wearing, suggesting hypoxia might correlate to AK induction. Further investigation of *Acanthamoeba* intracellular microbes will be necessary and may provide more information for the differential diagnostic and prognostic evaluation of AK.

## 4. Materials and Methods

### 4.1. Culture of *Acanthamoeba* Protozoa

The standard isolates ATCC-30010 and ATCC-50492 were *Acanthamoeba castellanii* which were isolated from soil and human cornea with keratitis and purchased from ATCC (Manassas, VA, USA). The clinical isolates were collected from the corneal ulcers of patients who were diagnosed with AK in the Cheng Kung University Hospital [24] and are characterized in Table 1. The study was conducted according to the guidelines of the Declaration of Helsinki, and approved by the Institutional Review Board of National Cheng Kung University Hospital (Protocol code: A-BR-101-124; Date of approval: 9 November 2012). *Acanthamoeba* isolates were cultured in protease peptone-yeast extract-glucose (PYG) medium (pH 6.5) at 28 °C in cell culture flasks and maintained after Page’s modified Neff’s amoeba saline (PAS: 1.2 g NaCl, 0.04 g MgSO_4_×7H_2_O, 0.03 g CaCl_2_, 1.42 g Na_2_HPO_4_, 1.36 g KH_2_PO_4_ in 1 L ddH_2_O) washing.

### 4.2. Genomic DNA Extraction and Sample Preparation

To identify the endosymbionts in *A. castellanii*, protozoan cells were grown in axenic culture and subsequently harvested. The cell pellets were suspended in PAS for genome extraction. DNA extraction was performed using a LabPrep DNA Mini kit^®^ (TAIGEN Bioscience Corporation, Taipei, Taiwan) according to the protocol described in the package insert.

### 4.3. 16S Ribosomal RNA Sequencing

The enriched DNA samples were then subjected to PCR reactions with specified forward (TCGTCGGCAGCGTCAGATGTGTATAAGAGACAGCCTACGGGNGGCWGCA G) and reverse (GTCTCGTGGGCTCGGAGATGTGTATAAGAGACAGGAC TACHVG GGTATCTAATCC) primers [41] to amplify the V3–V4 genomics region of bacterial 16S rRNA genes. To tested axenic cultures, the unused PYG medium and the medium from cultured *Acanthamoeba* were analyzed by polymerase chain reaction (PCR) (10 min at 95 °C, followed by 25 cycles of 30 secs at 95 °C, 30 secs at 55 °C, and 30 secs at 72 °C). To tested intracellular symbionts presenting, all the extracted DNA of isolates were analyzed by PCR as mentioned above. With approximately 550 bp PCR product, as confirmed by gel electrophoresis, the DNA samples were further subjected to sample preparation for metagenomics sequencing. DNA samples were prepared according to the 16S Metagenomics Sequencing Library Preparation instructions (Illumina, California, USA). The prepared amplicons were sequenced on a MiSeq instrument (Illumina, California, USA) using a 600-cycle sequencing reagent and with the specified paired-end mode.

### 4.4. Data Analyses

**Figure 6:**
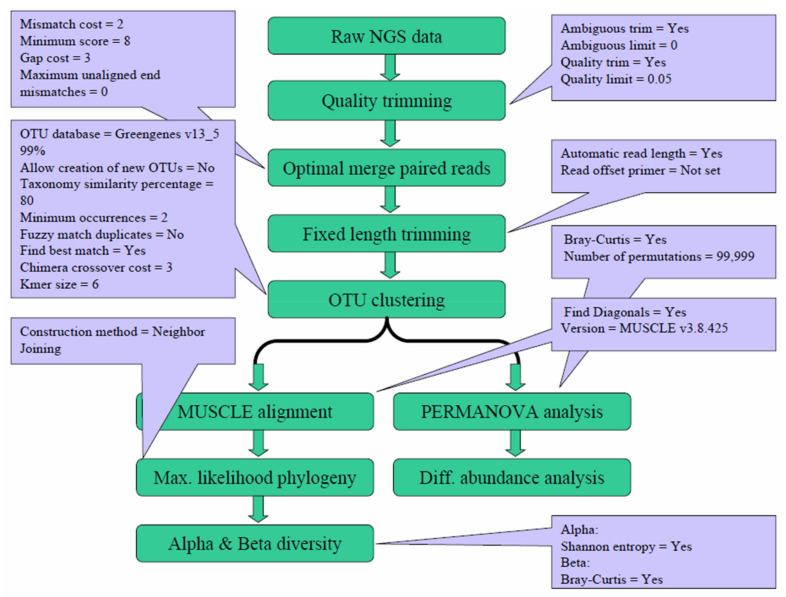
Workflow and parameters used for data analysis. The generated Next-generation sequencing (NGS) reads were subjected to quality trimming, merging, and fixed-length trimming, followed by operational taxonomic unit (OTU) clustering. The generated OTU table was analyzed for alpha and beta diversity. The parameters for each step are shown in the corresponding dialogue boxes.

The generated Next-generation sequencing (NGS) data were analyzed with the Microbial Genomics Module of CLC Genomics Workbench 9.5.4 (Qiagen, Stockach, Germany). As shown in Figure 6, the raw NGS reads were first subjected to quality trimming from the 3′ end, an optimal merging of paired reads, and fixed-length trimming, followed by operational taxonomic unit (OTU) clustering. After the OTU clustering step, the generated OTU table was further analyzed to calculate the alpha and beta diversity. Meanwhile, the generated OTU table was also subject to PERMANOVA analysis and differential abundance analysis. The parameters for each step were mainly the default values, with the specific values shown in the corresponding dialogue boxes.

### 4.5. Gene Abundance Comparison

The comparison of intracellular bacteria gene abundance was analyzed from the NGS database. The analyzed targets were classified into keratitis, soil, early-stage AK, and late-stage AK. Keratitis includes ATCC-50492, NCKUH-A, -B, -C, -D, and –H; soil includes ATCC-30010; early-stage AK includes NCKUH-A, -B, and –C; late-stage AK includes NCKUH-D and –H. To characterize the intracellular microbiome relative abundance ratio, gene expression fold change of keratitis was divided by soil and late-stage AK was divided by early-stage AK. The significant bacteria showed consistent abundance in the comparison within family, genus, and species.

## Figures and Tables

**Table 1:** Characteristics of *Acanthamoeba castellanii* isolates used in the study. Profiles for isolates ATCC-30010 and ATCC-50492 are as described on the American Type Culture Collection (ATCC^®^) official website. The A, B, and C strains were isolated from early keratitis patients. The D and H strains were isolated from keratitis patients treated with surgery at late progression.

Profile of *A. Castellanii* Strains in the Study			
Isolate	Progression	Source	Geosphere
ATCC-30010	N/A *	Environment	USA
ATCC-50492	Unknown^†^	Keratitis	India
NCKUH-A	Early	Keratitis	Taiwan
NCKUH-B	Early	Keratitis	Taiwan
NCKUH-C	Early	Keratitis	Taiwan
NCKUH-D	Late	Keratitis	Taiwan
NCKUH-H	Late	Keratitis	Taiwan

* Environmental strain with no pathogenic report express in N/A. † No progression data.
